# *Stylogaster* eggs on blow flies attracted to millipede defence secretions in Tanzania, with a stab at summarising their biology (Diptera: Conopidae & Calliphoridae)

**DOI:** 10.3897/BDJ.8.e54808

**Published:** 2020-06-30

**Authors:** Arn Rytter Jensen, Freja Odgaard, Pierfilippo Cerretti, Thomas Pape

**Affiliations:** 1 Sapienza University of Rome, Rome, Italy Sapienza University of Rome Rome Italy; 2 Natural History Museum of Denmark, University of Copenhagen, Copenhagen, Denmark Natural History Museum of Denmark, University of Copenhagen Copenhagen Denmark

**Keywords:** Afrotropical, Tanzania, Diptera, Conopidae, *
Stylogaster
*, Calliphoridae, dart-eggs, hosts, parasitoids, egg-carriers

## Abstract

The genus *Stylogaster* Macquart (Diptera: Conopidae) is sister to the remainder of the Conopidae. While all other Conopidae are endoparasitoids of aculeate Hymenoptera, species of *Stylogaster* appear to be endoparasitoids of ‘orthopteroids’, as the only confirmed rearing records are from crickets and cockroaches. Many calyptrate flies have been observed with *Stylogaster* eggs attached, but since no *Stylogaster* have been reared from any dipterans, it is still unknown if these flies are hosts, results of accidental oviposition or carry the eggs to the actual hosts. In this study, we report our findings of *Stylogaster* eggs on blow flies (Calliphoridae) attracted to millipede defence secretions in Tanzania. Out of seven different species collected and a total of 301 specimens, only flies of the genus *Tricyclea* Wulp had *Stylogaster* eggs attached. Out of 133 *Tricyclea* collected, 32 (24%) had *Stylogaster* eggs attached and, with one exception, all eggs were attached to the abdomen. The lifecycle of *Stylogaster* is summarised and discussed with a particular focus on dipteran egg-carriers.

## Introduction

The genus *Stylogaster* Macquart (Diptera: Conopidae) presents intriguing challenges with regard to the biology of its included species. The genus is remarkably distinct, with all species characterised by an extremely long, geniculate proboscis, elongate and tapering female terminalia and a harpoon-like anti-micropylar end of the egg (Brown et al. 2002). The genus has at times been placed in its own family (Stylogastridae, for example, by Séguy 1946, Rohdendorf 1964, Smith and Cunningham-Van Someren 1985), but has more recently been given subfamily status and recognised as sister taxon to the remainder of the Conopidae (Hennig 1966, McAlpine et al. 1989, Gibson et al. 2010, Gibson et al. 2012, Gibson and Skevington 2013). Currently, 125 species of *Stylogaster* are recognised in the world, with the main diversity in the Neotropical (73 species) and Afrotropical regions (42 species, including Madagascar), but species are also found in parts of North America, Asia, the Philippines, New Guinea, eastern Australia, Tasmania and New Caledonia (Smith 1967, Schneider 2010, Stuke 2012, Stuke 2017).

The natural history of *Stylogaster* is poorly understood and present evidence on the breeding biology is sparse, although host-seeking and apparently gravid females are frequently encountered (Rettenmeyer 1961a, Rettenmeyer 1961b, TP pers. obs.). Females of *Stylogaster* ram harpoon-like eggs into potential hosts, using their characteristic elongated oviscapt (Lopes 1937, Kotrba 1997) and present evidence indicates that the larvae are internal parasitoids, living in the abdomen of the host (Smith and Cunningham-Van Someren 1985, Woodley and Judd 1998, Etzler 2019). For the Nearctic *Stylogaster* with cricket hosts, the larva pupates outside of the host (Woodley and Judd 1998, Etzler et al. 2020) and the host usually dies upon emergence of the larva or lives for only a short time thereafter (Etzler et al. 2020). Many *Stylogaster* species are facultative army ant and driver ant followers (Carpenter 1914, Bequaert 1922, Bequaert 1930, Aldrich 1930, Lopes 1937, Cohic 1948, Rettenmeyer 1961a, Rettenmeyer 1961b). The females search for potential hosts in front of the leading edge of the advancing raids, looking for potential hosts fleeing from the ants (Stuckenberg 1963, Smith 1967, Smith 1969, Smith and Cunningham-Van Someren 1985, Kotrba 1997, Couri and Pont 2006, Couri and Barros 2010, PC pers. obs.). When not following army ants, adults of *Stylogaster* can be found hovering over sunlit paths in the forest understorey or feeding off the nectar on small white or yellow flowers (Rettenmeyer 1961a, Burt et al. 2014). The few hosts that are confirmed from actual rearing of *Stylogaster* specimens are all crickets and cockroaches (Smith and Cunningham-Van Someren 1985, Woodley and Judd 1998, Etzler et al. 2020). This stands in stark contrast to the fact that *Stylogaster* eggs have been found attached to several species of Diptera, none of which has been confirmed as hosts. These Diptera 'egg-carriers' are primarily calyptrate flies. In fact, there are only four observations of *Stylogaster* eggs on non-calyptrate flies, i.e. a single species (and specimen) each of Conopidae, Heleomyzidae, Lauxaniidae and Syrphidae (Berghe et al. 1956, Stuckenberg 1963, Smith and Cunningham-Van Someren 1985, Kotrba 1997). Rettenmeyer (1961a) was the first to mention *Stylogaster* ovipositing on Diptera, while studying army ants in Panama. He found *Stylogaster* eggs attached to tachinid flies of the genera *Calodexia* Wulp and *Phasia* Robineau-Desvoidy (as *Androeuryops* Beneway) following army ants and he observed *Stylogaster* ovipositing on unspecified “other insects”, but he provided no rearing records. Firm evidence of actual parasitisation is still restricted to Smith and Cunningham-Van Someren (1985), who dissected larvae of *Stylogaster* from immature cockroaches, as well as from one cricket in Kenya; Woodley and Judd (1998), who reported *Stylogaster biannulata* (Say) as reared repeatedly from *Gryllus rubens* Scudder in USA, Florida; and Etzler et al. (2020), who reared *Stylogaster neglecta* Williston from the cricket *Oecanthus nigricornis* (Walker) sampled from Canada (southern Ontario) and USA (New York State). The presence of *Stylogaster* eggs on various calyptrate flies has led to speculation about possible dipteran hosts, but as no larvae of *Stylogaster* have been found within any fly and no adults have been reared, this is still uncertain (Rettenmeyer 1961a, Rettenmeyer 1961b, Smith 1967, Smith 1969, Smith 1974, Smith and Cunningham-Van Someren 1985, Couri and Pont 2006, Couri and Barros 2010, Couri et al. 2013, Couri et al. 2019).

In this paper, we aim at compiling and reviewing available data on *Stylogaster* biology, with a focus on what is known about hosts and egg-carriers. We are adding our own data on *Stylogaster* eggs found on calyptrate flies from specific sampling focused on flies attracted to millipede defence secretions in Udzungwa Mountains National Park, Tanzania and we discuss the lifecycle of *Stylogaster* with its possible host range, in order to stimulate further research into the biology of *Stylogaster*.

## Material and methods

Specimens of potential egg-carriers were collected (by TP) by placing injured or crushed local juliform millipedes in a white plastic tray or on a sheet of white cloth. The flies attracted to the millipedes where collected using a hand net and stored in 70% ethanol for further examination. All collections where made on 21 August 2018 at the same locality in Tanzania: Mizimu camp, Udzungwa Mountains National Park, Morogoro Region, which is montane rainforest at an altitude of 769 m a.s.l. (07°48’23.40”S; 36°51’7.29”E). All material for this project has been deposited at the Natural History Museum of Denmark.

Specimens were examined and identified in 70% ethanol, with some being pinned and air-dried for imaging. For a reliable identification, male terminalia were dissected by making a cut between tergites 4 and 5, separating all of segment 5 plus the male terminalia and then isolating both sternite 5 and male terminalia from tergite 5. Male terminalia and sternite 5 were treated with 10% potassium hydroxide (KOH) for 24 hours at room temperature to macerate all soft tissue, then immersed in acetic acid, washed in distilled water, dehydrated in ethanol and transferred to glycerol for examination. After examination, all structures were stored in glycerol in a microvial pinned with the specimen. Each specimen with *Stylogaster* eggs was labelled with a unique identifier and the position of the eggs was recorded.

A series of photographs was taken using a Visionary Digital Imaging System with a Canon EOS 7D and stacked using Zerene Stacker version 2.0 (Zerene Systems LLC, Richland WA, USA). Superimposed photographs were edited using Adobe Photoshop® CS6 and GIMP 2.10. Drawings were digitally inked using Adobe Illustrator® CS6.

A thorough search was made in relevant literature for all records of *Stylogaster* hosts or egg-carriers and of other details of relevance for *Stylogaster* biology.

## Results

From the flies attracted to the wounded millipedes, a total of 301 calliphorid flies belonging to three different genera were collected and examined: *Phumosia* Robineau-Desvoidy (one species), *Hemigymnochaeta* Corti (four species) and *Tricyclea* Wulp (two species). Eggs of *Stylogaster* were only found on the two *Tricyclea* species: *Tricyclea fasciata* (Macquart) and *Tricyclea* sp. A, which were also the most numerous flies in the sample as 44% of the calliphorids belonged to *Tricyclea*. In total, 133 specimens of *Tricyclea* were collected, of which 32 (24%) had *Stylogaster* eggs attached and a total of 48 *Stylogaster* eggs were counted (Table 1). The number of eggs attached per individual ranged from one to five eggs, with an average of 1.4 eggs attached per individual. The distribution was as follows: 21 (44%) individuals with only one egg attached, nine (19%) with two eggs, zero with three eggs, one (2%) with four and one (2%) with five eggs attached. There was no significant difference in the number of eggs attached per individual between male and female flies (Fisher's Exact Test: *T. fasciata* (N = 23) *p* = 1, *T.* sp. A (N = 9) *p* = 0.57) or between the two *Tricyclea* species (Fisher's Exact Test: (N = 32) *p* = 0.6). *Stylogaster* eggs were predominantly found attached to female *Tricyclea*, although this was non-significant (Yates's chi-square (1, *N* = 165) = 0.28, *p* = 0.6), with 21 (65.6%) females and 11 (34.4%) males of the total of 32 *Tricyclea* individuals collected with eggs attached. However, there is a marked difference at the species-level, where more females carry eggs in *T. fasciata* (Yates's chi-square (1, *N* = 48) = 3.85, *p* = 0.05), while more males carry eggs in *T.* sp. A, although the latter difference is non-significant (Yates's chi-square (1, *N* = 117) = 0.17, *p* = 0.7).

**Figure 1.**
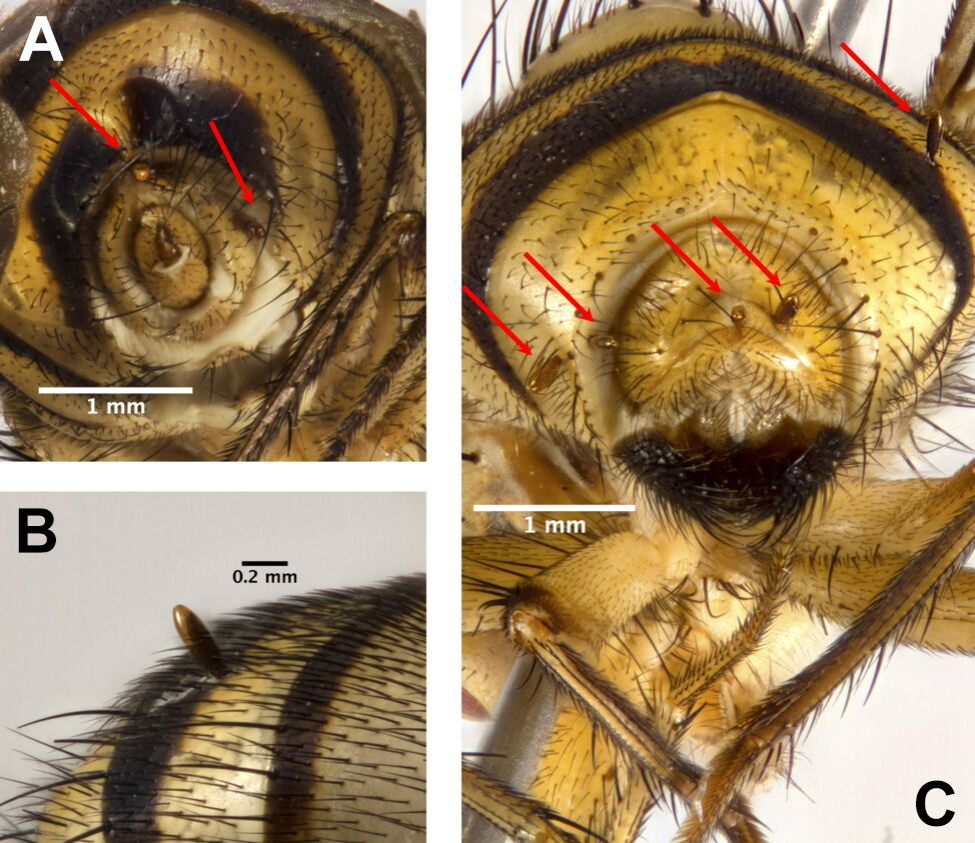
*Stylogaster* eggs found on *Tricyclea* spp. **A.** *Tricyclea fasciata* (Maquart), "Fly T", 2 eggs, posterior view; **B.** *Tricyclea* sp. A, "Fly J", egg on tergite 5; **C.** *Tricyclea* sp. A, "Fly J", 5 eggs, posterior view.

**Figure 2.**
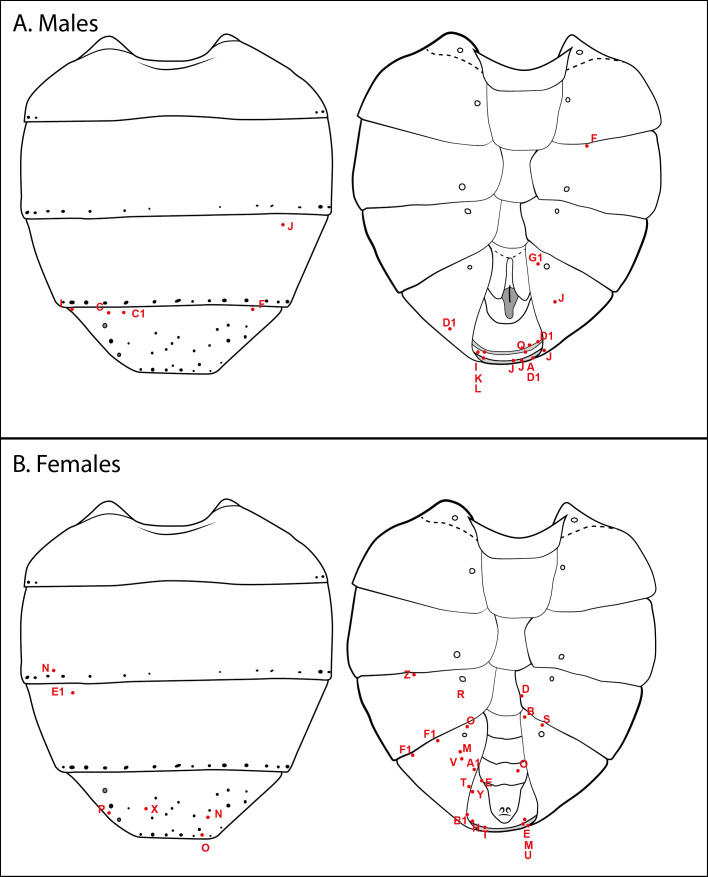
Position of *Stylogaster* eggs for all *Tricyclea* males (**A)** and females (**B**) collected. Red dots mark egg insertions and each specimen is denoted by an individual code, for example, there are two F1 as the specimen F1 had two eggs attached (see Suppl. material 1). Abdominal outlines modified from Rognes (1991) [figs. 10–11 of *Pollenia rudis* (Fabricius)].

*Stylogaster* eggs were only found attached to the posterior part of the abdomen of both male and female carriers (Fig. 1) and mostly to the ventral surface, with the single exception of an egg found on the thorax of a female, which also had one egg on the abdomen (Fly B1 in Fig. 2, Suppl. material 1). Eggs were most often attached to tergite 5 and the terminalia (Fig. 2, Suppl. material 1).

From our literature review, we found 268 observations of *Stylogaster* eggs on 68 different species of calyptrate flies (Table 2). Most of the observations are from the families Muscidae and Calliphoridae and these are all from the Afrotropical Region. The family Muscidae has 208 observations of flies with *Stylogaster* eggs attached, distributed on 15 genera and 48 species. The Calliphoridae have 48 observations, for three genera and 10 species. In the Neotropical region, the only records are from Tachinidae, with 17 observations for two genera and seven species. Around half of the calyptrate species recorded with *Stylogaster* eggs have more than one record per species and the eggs are primarily found on female flies (Table 2).

The *Stylogaster* species identified from attached eggs and host or egg-carrier data are compiled in Table 3. Number of *Stylogaster* eggs for specific body parts of calyptrate egg-carriers is summarszed for each genus in Table 4. Average of *Stylogaster* eggs per fly for calyptrate egg-carriers is presented in Table 5 and the proportion of calyptrate flies with *Stylogaster* eggs versus the total number of calyptrate flies collected is presented in Table 6.

## Discussion

### Known hosts of Stylogaster

The only hosts of *Stylogaster* that are confirmed from actual rearing records are cockroaches (Blattodea) and crickets (Orthoptera, Gryllidae) from the Nearctic and Afrotropics (Table 3). Three *Stylogaster* species have been reared from crickets: the Nearctic *S. biannulata* (Say) and *S. neglecta* Williston (Woodley and Judd 1998, Etzler et al. 2020) and the Afrotropical *S. westwoodi* Smith (Smith and Cunningham-Van Someren 1985). One species has been reared from cockroaches: the Afrotropical *S. varifrons* Malloch (Smith and Cunningham-Van Someren 1985). Besides these rearing records, there are only two other records of *Stylogaster* eggs attached to non-dipterans, which are those of Lopes (1937), who reported one egg attached to a cockroach (*Chorisoneura* sp.) and one egg attached to an undetermined orthopteran. There has been some confusion about other records of eggs on crickets and cockroaches, i.e. Ferrar (1987) referring to Rettenmeyer (1961b) as confirmed records of eggs and Taber and Maloney (2006) referring to Stuckenberg (1963), but in both cases, these works do not provide any new records and refer to the records from Lopes (1937). The almost total lack of records of *Stylogaster* eggs attached to cockroaches and crickets, as compared to the numerous records of eggs on calyptrate flies, could be due to a lack of coordinated search efforts, as suggested by the study of Etzler et al. (2020), where many crickets with *Stylogaster* eggs and larvae were collected after targeted sampling. Another explanation could be that eggs have been overlooked or that eggs detach after some time, as one observation suggests (Smith and Cunningham-Van Someren 1985). An explanation could also be that hosts are induced to express a ‘grave-digging’ behaviour before dying, as has been documented for larvae of some Conopidae in their hymenopteran hosts (Müller 1994, Rasmussen and Cameron 2004, Malfi et al. 2014), which would make parasitised hosts less prone to being collected.

### Stylogaster egg-carriers

Stylogaster eggs have been found attached to several different dipterans of the families Anthomyiidae, Calliphoridae, Heleomyzidae, Lauxaniidae, Muscidae, Rhiniidae, Syrphidae, Tachinidae, even Conopidae (a *Stylogaster*!) and eggs have also been found on a spider (Table 2). The non-calyptrate records are all single specimens and could easily be explained away as accidental egg-impaling by *Stylogaster* females. In contrast to this, a large range of calyptrate flies have many records of *Stylogaster* eggs, especially species of Muscidae, Calliphoridae and Tachinidae, some even from the same collection event (Table 6). However, despite the presence of eggs, no *Stylogaster* larva has ever been recovered inside a calyptrate fly; this could be due a lack of a coordinated effort of dissecting the flies carrying the eggs, as all authors remove the *Stylogaster* eggs without dissection of the carrier flies. The only attempt at dissecting known egg-carrier flies is by Rettenmeyer (1961a), who dissected 20 females of *Calodexia* without *Stylogaster* eggs, but with abnormal abdomens and without finding any *Stylogaster* larvae. Therefore, there is no firm evidence that these flies are regular or occasional hosts or even if they are hosts at all. It has been speculated that the calyptrate flies instead are used to transport the eggs to the final host or food source or that the flies just happen to share the same appearance or habitat as the host of *Stylogaster* and, therefore, accidentally become impaled with eggs (Smith 1967, Ferrar 1987, Couri and Pont 2006, Couri and Barros 2010, Stuke 2012, Couri et al. 2013), but the evidence to support this remains circumstantial.

As noted by Stuckenberg (1963), the Afrotropical egg-carriers seem to be mostly yellowish-brown and forest dwelling. Almost all the dipteran egg-carriers share a yellowish-brown abdomen with black or dark stripes (Table 2). This could indicate that this pattern somehow triggers *Stylogaster* to oviposit, either because the flies are potential hosts or because they resemble the actual *Stylogaster* host. However, the crickets and cockroaches, so far recorded as hosts, do not have this pattern on their abdomen.

### Stylogaster egg placement

Females of *Stylogaster* predominantly attach their eggs to the abdomen of the host (Lopes 1937, Smith and Cunningham-Van Someren 1985, Woodley and Judd 1998, Etzler 2019) and only eggs attached to the abdomen develop successfully (Etzler 2019). This, combined with the fact that the *Stylogaster* larva, as with other Conopidae (Brown et al. 2002, Stuke 2017), develops in the abdomen of the host, would indicate that the eggs must be attached to the host abdomen for a successful development and that eggs attached elsewhere would be misplaced and unsuccessful.

The distribution of *Stylogaster* eggs on the calyptrate flies appears to vary between genera (data too sparse to allow assessment per species). Taking into consideration the different proportions of surface area for head (17.5%), thorax (54.1%) and abdomen (28.4%), Stuckenberg (1963) and Smith (1967) found the attached *Stylogaster* eggs to be randomly distributed on the bodies of the flies, for example, from the muscid genera *Dichaetomyia* Malloch, *Dimorphia* Malloch and *Pyrellina* Malloch. This agrees reasonably well with our data compiled for Muscidae, where most eggs are placed on the thorax (62%), followed by the abdomen (19%) and head (15%) (Table 4). However, the Calliphoridae and Tachinidae have most *Stylogaster* eggs attached to the abdomen, with 89% and 65%, respectively (Table 4). The present material of *Tricyclea* spp. shows a strong concentration of *Stylogaster* eggs on the postero-ventral part of the abdomen, which would make sense if the *Stylogaster* female attacks a flying potential carrier from behind. This is also in agreement with Rettenmeyer (1961a), who reported *Stylogaster* eggs to be concentrated on the abdomen of females of *Calodexia* spp. and one male and female of *Phasia ecitonis* (Table 4). The known Neotropical carriers are almost exclusively female Tachinidae with a host-seeking behaviour associated with foraging army ants and with hosts amongst Orthoptera, Blattodea and Heteroptera, which are attacked as they flee from the ants (Rettenmeyer 1961a, Rettenmeyer 1961b, Brown et al. 2010). If species of *Stylogaster* share one or more hosts with species of *Calodexia*, a possible scenario would be *Stylogaster* females accidentally impaling females of *Calodexia* when both are darting after an orthopteran or a cockroach fleeing from the foraging ants.

### Oviposition strategy

Parasitoids, like *Stylogaster*, with a direct deposition strategy, produce a small number of eggs and often tend to be oligo- or monophagous. The clutch size of the Afrotropical species of *Stylogaster* is about 60-128 eggs (Stuckenberg 1963, Smith 1967), the Neotropical *S. stylosa* carries 120 eggs (Kotrba 1997) and the Neartic *S. neglecta* carries the most eggs with around 155 eggs per female (Taber and Maloney 2006). The modest number of eggs is likely related to a high rate of successful parasitisations, i.e. the gravid female allocates more energy to host seeking and egg deposition in order to secure the offspring an optimal developmental environment, rather than to increased egg production, as is common in parasitoids with an indirect oviposition strategy, where eggs have a lower chance of being picked up by a suitable host. It would, therefore, appear likely that *Stylogaster* females could afford to ‘waste’ only very few eggs on non-host impaling. Etzler (2019) observed that mature *Stylogaster* larvae sharing a host appeared to be smaller than larvae that did not and only a few crickets had multiple larvae. This indicates that one or only a few eggs per host is the optimal strategy, as smaller larvae from shared hosts would produce smaller adult flies with a lower fitness (Etzler 2019). However, this could vary with host size, as the two cockroaches examined by Smith and Cunningham-Van Someren (1985) both had multiple eggs and larvae. Calyptrates with many observations of *Stylogaster* eggs show an average number of 1.45 eggs per fly (Table 5). This compares with 1.25 *Stylogaster* larvae per cricket found by Etzler (2019), who does not provide an estimate of number of eggs per cricket.

### Larval biology

**Figure 3.**
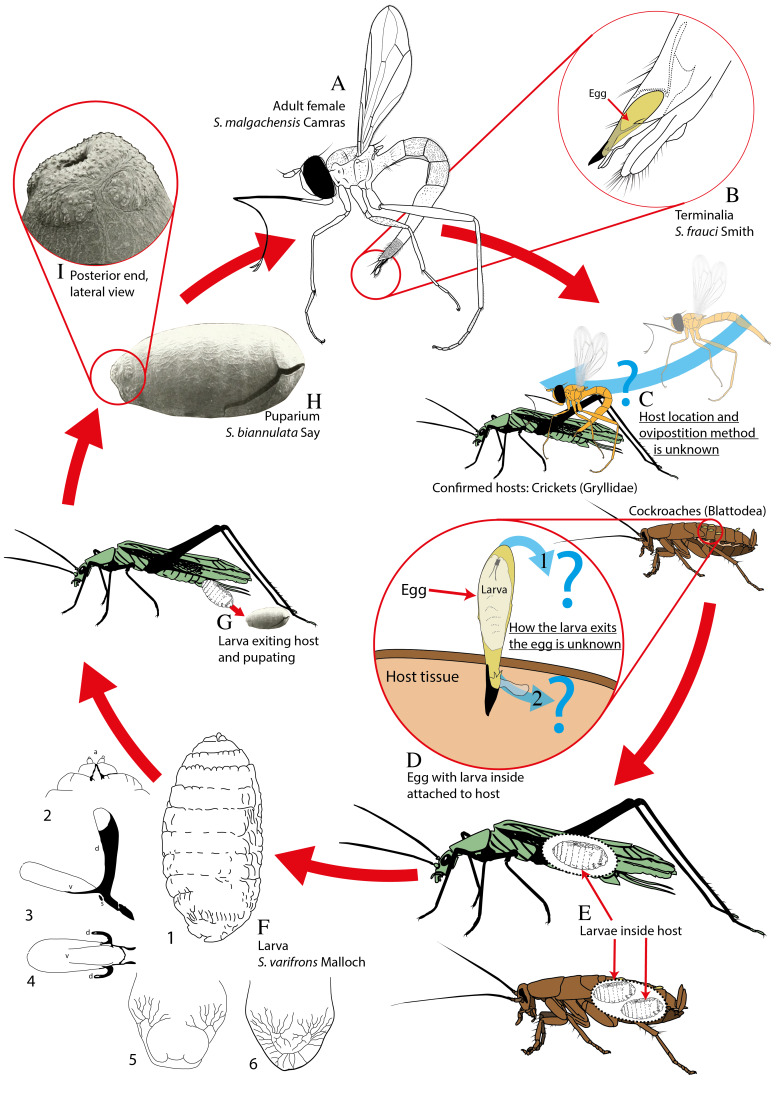
Lifecycle of *Stylogaster*. **A.** Adult female of *S. malgachensis* Camras; notice the bent abdomen which the females flicks when hovering in flight (Rettenmeyer 1961a, Kotrba 1997); **B.** Close-up of terminalia from *S. frauci* Smith. Egg lodged in terminal chamber with anti-micropylar end protruding; **C.** Host location and ovipostition method unknown (Kotrba 1997). Confirmed hosts: Crickets (Gryllidae) and cockroaches (Blattodea); **D.** Egg attached to host. Anti-micropylar end is inside the host, extrusible sac and spines keeping the egg from falling off (**2**). How the larva exits the egg is unknown, the two proposed ways are illustrated (**1** and **2**); **E.** Larvae developing inside hosts; **F.** Illustration of *Stylogaster* larva, *S. varifrons* Malloch (Smith and Cunningham-Van Someren 1985). **1** Whole larva in left lateral view. **2** Ventral view of anterior end showing antennae and mouthparts. **3** Cephalopharyngeal skeleton in lateral view and **4** dorsal view. **5** Posterior end of larva showing network of tracheoles in ventral view and **6** dorsal view; **G.** Larva exiting host from the end of the abdomen and pupating (Etzler et al. 2020); **H.** SEM of *Stylogaster* puparium, *S. biannulata* (Say) and close-up of **I.** posterior end, lateral view (Woodley and Judd 1998). The method of oviposition (**C**) and how the larva exits from the egg and enters the host (**D**) are still unknown, shown by blue arrows and question marks (?). Compiled from literature and based on (**A, C**) *S. malgachensis* Camras, (**B, D**) *S. frauci* Smith, (**E, F, G**) *S. varifrons* Malloch and (**G, H, I**) *S. biannulata* (Say), as all life stages from a single species were not available. Modified from Smith (1967), Smith and Cunningham-Van Someren (1985), Kotrba (1997), Woodley and Judd (1998).

It is not known how the *Stylogaster* larva enters the host (Fig. 3). Some have suggested that the larva enters through the extrusible sac at the anti-micropylar end (Fig. 3D2), as the barbed part of the egg, which is stabbed into the host, is presumed too heavily sclerotised for the larva to exit. Other records indicate that the larva may emerge from the blunt micropylar end of the egg, facing away from the host (Fig. 3D1, Rettenmeyer 1961a, Rettenmeyer 1961b, Stuckenberg 1963). This is further supported by records of larvae inside attached eggs, placed with their head towards the blunt micropylar end of the egg (Fig. 3D, Smith and Cunningham-Van Someren 1985, Couri et al. 2013), and Stuckenberg (1963) found an empty egg attached to *Dichaetomyia quadrata* (Wiedemann) with the micropylar end "irregularly broken open" and interpreted this as a hatched egg. If the larva hatches from the egg through the end facing away from the host, especially for eggs that are attached to the ventral part of the host, it would appear that there is a high risk that the emerging larva would fall off the host. That would support the hypothesis of the calyptrate flies functioning as egg-carriers rather than true hosts.

### Biology of hosts/egg-carriers

The two species of *Tricyclea* Wulp found to be impaled by *Stylogaster* eggs in the present study have a remarkably similar – and notably high – rate of infection (23–24%) (Table 1). This matches the overall infection rate of the cricket *Oecanthus nigricornis*, which is the known host of the Nearctic *S. neglecta*, although the infection rate for individual crickets can vary significantly per site (Table 6, Etzler et al. 2020). Male crickets of medium size had a significantly higher rate of parasitism than females in the Etzler (2019) study. Amongst the calyptrate flies, *Stylogaster* eggs were predominantly attached to female flies, although this could be an artifact of limited sampling as most species have very few records and many records are from different collection events (Table 2) or it may be due to a higher abundance of female flies where *Stylogaster* search for hosts. More surprisingly, while specimens of both (and therefore all) species of *Tricyclea* were impaled, none of the four species of *Hemigymnochaeta* Corti was impaled (Table 1), even though all six species are very similar (at least to a human observer) and were collected at the same event. Other studies have recovered specimens of *Hemigymnochaeta* carrying eggs of *Stylogaster* (Table 2).

Species of *Tricyclea* and *Hemigymnochaeta* are practically unknown biologically, although there are indications that they are all associated with termite or ant nests, including the fruiting bodies of *Termitomyces* Heim emerging from nests of Macrotermitinae (Zumpt 1953). *Tricyclea evanida* Villeneuve and *T. fasciata* (Macquart) have been reared from the refuse piles of the ant *Paltothyreus tarsatus* (Fabricius) and *T. semithoracica* Villeneuve and *T. perpendicularis* Villeneuve have been observed ovipositing near the nests of driver ants (*Dorylus* Fabricius) (Villeneuve 1922). The flies included in the present study were collected as they were attracted to the benzoquinone-based defence secretions of juliform millipedes (T. Pape, pers. obs.), which is behaviour known from the millipede-associated species of the Nearctic flesh fly genus *Spirobolomyia* Townsend and probably the pantropical scuttle fly genus *Myriophora* Brown (Hash et al. 2017, Hash et al. 2018), but not previously documented for blow flies. A possible explanation could be that foraging driver ants encountering millipedes will cause the latter to release their defence secretions, which will attract a variety of flies (Table 1) and which are then coming into the range of host-seeking *Stylogaster* females hunting for hosts. This would then be similar to the case of Neotropical Tachinidae with *Stylogaster* eggs, which have host-seeking behaviour associated with foraging army ants and a similar pattern of *Stylogaster* eggs attached on the abdomen as seen in *Tricyclea*, although the proportion of the Tachinidae with *Stylogaster* eggs, *Calodexia* spp. at 0.8% and *Phasia ecitonis* at 0.3%, is much lower than that reported here for *Tricyclea* (24%) (Table 6, Rettenmeyer 1961a).

This will not, however, explain why the species of *Tricyclea* have *Stylogaster* eggs predominantly inserted at the tip of the abdomen rather than distributed randomly as for other calyptrate flies, nor will it explain why, in the material studied here, species of *Tricyclea* are impaled, while those of *Hemigymnochaeta* are not.

### Phylogenetics and biogeography

**Figure 4.**
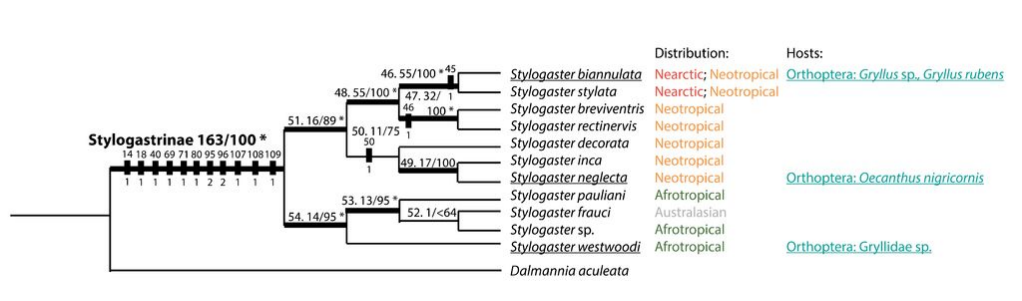
Phylogenetic tree modified from Gibson et al. (2012). Most-parsimonious cladogram generated from combined molecular and morphological data, see Gibson et al. (2012) for details. Host and biogeographical information added. *Stylogaster* with confirmed hosts underlined. Biogeographical information from Stuke (2017) and host information from Table 3.

Due to our limited data on *Stylogaster* hosts, there seems to be no phylogenetic pattern in the position of *Stylogaster* with confirmed hosts. *Stylogaster* species that parasitise crickets are found in all three major *Stylogaster* groups and both in the Nearctic, Neotropics and Afrotropics (Fig. 4). The pattern seems to be the same for *Stylogaster* parasitising cockroaches. The only rearing record is from the Afrotropical *S. varifrons*, but *Stylogaster* eggs have also been found on cockroaches from two *Stylogaster* species with distributions in the Nearctic and the Neotropics. The same holds true for the records of *Stylogaster* species with eggs on dipterans, which are also found in both the Nearctic, Neotropics and Afrotropics, although it is noteworthy that the majority of records – and all the non-tachinids – are from the Afrotropics (Table 2).

### Conclusion

*Tricyclea fasciata* and *T.* sp. A appear to be likely candidates for dipteran hosts of *Stylogaster*, even though a rearing record is still needed to finally confirm this. The records of *Stylogaster* eggs on *Tricyclea* differ from those from other calyptrates and support the hypothesis that species of *Tricyclea* are hosts of *Stylogaster*. First, the proportion of *Tricyclea* with *Stylogaster* eggs reported here (24%) is higher than most of the other calyptrate observations. Second, the *Stylogaster* egg placement on the abdomen of *Tricyclea* is similar to that on the confirmed hosts of *Stylogaster* and not random as for most of the other calyptrates. Third, the morphologically very similar *Hemigymnochaeta* that were collected from the same site as the egg-carrying *Tricyclea* spp. had no *Stylogaster* eggs, which suggests targeted rather than indiscriminate oviposition.

We are getting closer to understanding the biology of *Stylogaster* (Fig. 3), but there are still some major questions left. First and foremost, there is a need for hosts confirmed through rearing, which will also bring an indication of the host range. We need more data on the oviposition behaviour of *Stylogaster* females and, in particular, on how the larva hatches from the egg and enters its host.

Answering these questions is crucial if we want to understand the complex biological interactions that *Stylogaster* is a part of and the early evolution of Conopidae. For example, if host location is mainly by visual cues, looking for patterns or movement, as the observations of *Stylogaster* darting at moving hosts near army ants would indicate, then that would explain the association of *Stylogaster* with army ants and the eggs impaled in Neotropical *Calodexia* and, possibly, also why eggs are found predominantly in Afrotropical calyptrates with similarly-coloured abdomen.

## Supplementary Material

DFA851C0-2DA7-5C3C-872B-ECADC8C49106 10.3897/BDJ.8.e54808.suppl1 Supplementary material 1 Supplementary Table S1 Data type morphological Brief description Data on the placement and number of *Stylogaster* eggs for each individual fly examined. The ID of the fly corresponds to the visual representation of this data in Fig. 2. File: oo_411417.pdf https://binary.pensoft.net/file/411417 Arn Rytter Jensen, Freja Odgaard, Pierfilippo Cerretti, Thomas Pape

## Figures and Tables

**Table 1.** Number of specimens pr. species collected from dead or injured millipedes and the proportions with eggs of *Stylogaster* (Tanzania: Morogoro Region, Udzungwa Mountains National Park, Mizimu camp, 769 m a.s.l., 07°48’23.40”S; 36°51’7.29”E).

**Number of specimens collected per species**				***Stylogaster* eggs on carriers according to sex**		
**Species**	**Males**	**Females**	**Total**	**Males**	**Females**	**Total**
*Phumosia chukanella* Lehrer	14	24	38	-	-	-
*Tricyclea fasciata* (Macquart)	29	10	39	3 (10.3%)	6 (60.0%)	9 (23.0%)
*Tricyclea* sp. A	26	68	94	8 (30.8%)	15 (22.1%)	23 (24.4%)
*Hemigymnochaeta unicolor* Bigot	24	45	69	-	-	-
*Hemigymnochaeta gogoiana* Lehrer	8	28	36	-	-	-
*Hemigymnochaeta dashaniella* Lehrer	3	1	4	-	-	-
*Hemigymnochaeta* sp. A	10	11	21	-	-	-
**Total**	114	187	301	n/a	n/a	n/a

**Table 2.** Records of *Stylogaster* egg-carriers and hosts worldwide. Confirmed hosts with rearing records are underlined. Obs. = total number of observations for a given carrier species. For each egg-carrier the sex is given followed by the number of observations, for example, ♀:3 ♂:1 ?:1 for three females, one male and one specimen of unknown sex with one or more *Stylogaster* eggs attached. Observations are a total of all observations from references given. For taxa not identified to species level, the taxa follow the original identification and are put in quotations marks, for example, 'Lauxanidae sp.' from Stuckenberg (1963). [*Misspelled as “*Suilla* sp”. †Given as “*Dichaetomyia albinita* Stein”. ☼ Given as ”*Pseudobdellia subsetosa* Curran”, here interpreted as *Helina subsetosa* Curran, 1938, with the valid name *Pseudohelina nigritarsis* (Jaennicke, 1867).]

**Egg-carrier or host**		**Obs.**		**Region**		**Country**	**Reference**	
** ARANEAE **								
Lycosidae sp.		No data		Afrotropical		Kenya	Smith and Cunningham-Van Someren 1985	
** DIPTERA **								
** CONOPIDAE **								
*Stylogaster stylosa* Townsend, 1897		♀:1		Neotropical		Costa Rica	Kotrba 1997	
** HELEOMYZIDAE **								
Suillia cf. acroleuca (Speiser, 1910)		?:1		Afrotropical		Nigeria	Smith and Cunningham-Van Someren 1985*	
** SYRPHIDAE **								
*Asarkina hulleyi* Munro, 1924		♂:1		Afrotropical		Mozambique	Stuckenberg 1963	
** LAUXANIIDAE **								
Lauxanidae sp.		♂:1		Afrotropical		Madagascar	Stuckenberg 1963	
** GLOSSINIDAE **								
*Glossina morsitans* Westwood, 1851		?		Afrotropical		Rwanda	Berghe et al. 1956	
** MUSCIDAE **								
*Afromydaea debilis* (Stein, 1913)		♀:1		Afrotropical		South Africa	Couri et al. 2013	
*Coenosia ruwenzorica* (Emden, 1940)		♂:1		Afrotropical		Burundi	Couri et al. 2013	
*Deltotus facetus* Séguy, 1935		♀:3 ♂:1		Afrotropical		Madagascar	Couri and Pont 2006, Couri and Barros 2010	
*Deltotus viola* Zielke, 1972		♀:2		Afrotropical		Madagascar	Couri and Pont 2006	
Dichaetomyia (Panaga) sp. 1		♂:1		Afrotropical		Burundi	Couri et al. 2013	
Dichaetomyia (Panaga) sp. 2		♀:1 ♂:1		Afrotropical		South Africa	Couri and Barros 2010, Couri et al. 2013	
*Dichaetomyia albivitta* (Stein, 1906)		?:1		Afrotropical		Kenya	Smith and Cunningham-Van Someren 1985†	
*Dichaetomyia apicalis* (Zielke, 1972)		♀:1		Afrotropical		Madagascar	Couri and Pont 2006	
*Dichaetomyia basilaris* (Zielke, 1972)		♂:1		Afrotropical		Madagascar	Couri and Pont 2006	
*Dichaetomyia distanti* Malloch, 1921		?:1		Afrotropical		Kenya	Smith and Cunningham-Van Someren 1985	
*Dichaetomyia immaculiventris* Malloch, 1930		?:1		Afrotropical		Ethiopia	Smith and Cunningham-Van Someren 1985	
Dichaetomyia n. sp. cf. mallochi Emden, 1942		♀:3		Afrotropical		South Africa	Stuckenberg 1963	
*Dichaetomyia pallidula* Curran, 1935		?:1		Afrotropical		Ethiopia	Smith and Cunningham-Van Someren 1985	
*Dichaetomyia quadrata* Wiedemann, 1824		♀:1		Afrotropical		Mozambique	Stuckenberg 1963	
*Dichaetomyia serena* Stein, 1906		♀:2		Afrotropical		South Africa	Stuckenberg 1963	
*Dichaetomyia* sp. 1		♀:5 ♂:2		Afrotropical		Madagascar	Smith 1967	
*Dichaetomyia* sp. 2		♀:5 ♂:6		Afrotropical		Madagascar	Smith 1967	
*Dichaetomyia tristis* (Zielke, 1972)		♀:1 ♂:1 ?:1		Afrotropical		Madagascar Kenya	Smith and Cunningham-Van Someren 1985, Couri and Pont 2006	
*Dimorphia setulosa* Stein, 1918		♀:5		Afrotropical		South Africa	Stuckenberg 1963	
*Dimorphia tristis* Wiedemann, 1819		♀:9		Afrotropical		South Africa	Stuckenberg 1963, Couri et al. 2013	
*Haematobosca praedatrix* (Enderlein, 1928)		♀:2 ♂:2		Afrotropical		Uganda	Smith 1969	
*Hebecnema semiflava* Stein, 1913		♀:1		Afrotropical		Burundi	Couri et al. 2013	
*Helina carpiae* Couri, Pont & Penny, 2006		♀:1		Afrotropical		Madagascar	Couri and Barros 2010	
*Helina grisella* Couri, Pont & Penny, 2006		♀:1		Afrotropical		Madagascar	Couri and Barros 2010	
*Helina pervittata* Emden, 1951		♀:5		Afrotropical		Kenya	Smith 1969	
*Helina* sp.		♂:1		Afrotropical		South Africa	Couri et al. 2013	
*Limnophora obsignata* (Rondani, 1866)		♀:1		Afrotropical		Burundi	Couri et al. 2013	
*Limnophora translucida* Stein, 1913		♂:1		Afrotropical		Ethiopia	Couri et al. 2019	
*Musca lusoria* Wiedemann, 1824		♀:3		Afrotropical		Ethiopia	Couri et al. 2019	
*Musca splendens* Pont, 1980		♀:1		Afrotropical		Ethiopia	Couri et al. 2019	
*Neomyia chrysopyga* (Emden, 1939)		♀:1		Afrotropical		Ethiopia	Couri et al. 2019	
*Neomyia setulosa* (Zielke, 1972)		♀:1		Afrotropical		Madagascar	Couri and Pont 2006	
*Phaonia abnormis* Stein, 1906		♂:1		Afrotropical		Nigeria	Smith 1969	
*Phaonia plurivittata* Couri, Pont & Penny, 2006		♂:1		Afrotropical		Madagascar	Couri and Pont 2006	
*Phaonia* sp.		?:1		Afrotropical		Ethiopia	Smith and Cunningham-Van Someren 1985	
*Pseudohelina nigritarsis* (Jaennicke, 1867)		♀:2 ♂:3 ?:1		Afrotropical		Burundi; Ethiopia; Kenya	Couri et al. 2013, Couri et al. 2019, Smith and Cunningham-Van Someren 1985☼	
*Pseudohelina phaeoxantha* (Emden, 1951)		♀:1		Afrotropical		Burundi	Couri et al. 2013	
*Pseudohelina* sp. 1		♀:1		Afrotropical		Burundi	Couri et al. 2013	
*Pseudohelina* sp. 2		♀:1		Afrotropical		Kenya	Couri et al. 2013	
*Pyrellina abdominalis* Zielke, 1971		♀:9		Afrotropical		Burundi	Couri et al. 2013	
*Pyrellina chrysotelus* (Walker, 1853)		♀:1		Afrotropical		South Africa	Stuckenberg 1963	
*Pyrellina versatilis* (Villemeuve, 1916)		♀:2		Afrotropical		Burundi	Couri et al. 2013	
*Stomoxys brunnipes* Grunberg, 1906		♀:8		Afrotropical		Uganda	Smith 1969	
*Stomoxys inornata* Grunberg 1906		♀:6		Afrotropical		Uganda	Smith 1969	
*Stomoxys ochrosoma* Speiser, 1910		♀:1		Afrotropical		Kenya	Smith 1969	
*Stomoxys omega* Newstead, 1907		♀:66 ♂:7		Afrotropical		Ethiopia	Smith 1969, Couri et al. 2019	
*Stomoxys taeniatus* Bigot, 1888		♀:6 ♂:2		Afrotropical		Ethiopia	Couri et al. 2019	
*Stomoxys varipes* (Bezzi, 1907)		♀:1		Afrotropical		Ethiopia	Couri et al. 2019	
** ANTHOMYIIDAE **								
*Emmesomyia* sp.		♀:2		Afrotropical		Nigeria	Smith 1969	
** CALLIPHORIDAE **								
*Bengalia depressa* Walker, 1858		♂:2		Afrotropical		Kenya Zimbabwe	Smith 1969	
*Bengalia floccosa* (Wulp, 1884)		?:1		Afrotropical		Kenya	Smith and Cunningham-Van Someren 1985	
*Bengalia peuhi* Villeneuve, 1914		?:1		Afrotropical		Kenya	Smith and Cunningham-Van Someren 1985	
*Bengalia spinifemorata* Villeneuve, 1913		?:2		Afrotropical		Kenya	Smith and Cunningham-Van Someren 1985	
*Hemigymnochaeta unicolor* Bigot, 1888		♂:1 ?:2		Afrotropical		Nigeria Kenya	Smith 1969, Smith and Cunningham-Van Someren 1985	
*Hemigymnochaeta* sp.		♂:2		Afrotropical		Sierra Leone Cameroon	Smith 1969	
*Tricyclea bifrons* Malloch, 1929		?:2		Afrotropical		Kenya	Smith and Cunningham-Van Someren 1985	
*Tricyclea fasciata* Macquart, 1843		♀:6 ♂:3		Afrotropical		Tanzania	Present study	
*Tricyclea* n. sp.		♀:15 ♂:8		Afrotropical		Tanzania	Present study	
*Tricyclea* sp.		♂:3		Afrotropical		Uganda	Smith 1969	
** RHINIIDAE **								
Isomyia cf. pubera (Villeneuve, 1917)		?:1		Afrotropical		Kenya	Smith and Cunningham-Van Someren 1985	
** TACHINIDAE **								
*Phasia ecitonis* (Townsend, 1897)		♀:1 ♂:1		Neotropical		Panama	Rettenmeyer 1961a	
*Calodexia agilis* Curran, 1934		♀:7		Neotropical		Panama	Rettenmeyer 1961a	
*Calodexia dives* Curran, 1934		♀:3		Neotropical		Panama	Rettenmeyer 1961a	
*Calodexia fumosa* (Townsend, 1912)		♀:1		Neotropical		Panama	Rettenmeyer 1961a	
*Calodexia interrupta* Curran, 1934		♀:2		Neotropical		Panama	Rettenmeyer 1961a	
*Calodexia panamensis* (Townsend, 1919)		♀:1		Neotropical		Panama	Rettenmeyer 1961a	
*Calodexia venteris* Curran, 1934		♀:1		Neotropical		Panama	Rettenmeyer 1961a	
** ORTHOPTERA **								
*Acanthogryllus fortipes* (Walker, 1869)		No data		Afrotropical		Kenya	Smith and Cunningham-Van Someren 1985	
*Callogryllus* sp.		No data		Afrotropical		Kenya	Smith and Cunningham-Van Someren 1985	
Gryllidae sp.		No data		Afrotropical		Kenya	Smith and Cunningham-Van Someren 1985	
*Gryllus* sp.		No data		Nearctic		USA	Woodley and Judd 1998	
*Gryllus rubens* Scudder, 1902		No data		Nearctic		USA	Woodley and Judd 1998	
*Oecanthus nigricornis* Walker, 1869		No data		Nearctic		USA	Etzler et al. 2020	
Orthoptera sp.		No data		Neotropical		Brazil	Lopes 1937	
Pternoscirta cf. bimaculata Thunberg, 1815		No data		Afrotropical		Kenya	Smith and Cunningham-Van Someren 1985	
** BLATTODEA **								
Blattodea sp.		No data		Afrotropical		Kenya	Smith and Cunningham-Van Someren 1985	
Blattella cf. lobiventris (Saussure, 1895)		No data		Afrotropical		Kenya	Smith and Cunningham-Van Someren 1985	
*Chorisoneura* sp.		No data		Neotropical		Brazil	Lopes 1937	
Euloboptera cf. shelfordi Princes, 1955		No data		Afrotropical		Kenya	Smith and Cunningham-Van Someren 1985	

**Table 3.** The *Stylogaster* species identified from attached eggs and host or egg-carrier. References as in Table 2. For taxa not identified to species level, the taxa follow the original identification and are put in quotations marks, for example, 'S. cf. ornatipes Kröber, 1914' from Lopes (1937). [^α^Lopes (1937) did not find any *S. stylata* eggs attached to Blattodea, but mentioned several observations of attacks from *S. stylata.* ^β^Genus misspelled as “*Suilla*”. ^γ^Given as “*Dichaetomyia albinita* Stein”. ^δ^Given as ”*Pseudobdellia subsetosa* Curran”, here interpreted as *Helina subsetosa* Curran, 1938, with the valid name *Pseudohelina nigritarsis* (Jaennicke, 1867). ^ε^Smith and Cunningham-Van Someren (1985) observed *S. westwoodi* attacking cockroaches, but they did not find any eggs on the cockroach in which they found a *Stylogaster* larva. ^ζ^Smith (1967) stated that the Berghe et al. (1956) record of one male *S. leonum* reared from a pupa of *G. morsitans* is a misidentification of *S. westwoodi*.]

***Stylogaster* species**	**Host or egg-carrier**	**Region**	**Country**	**References**
*S. banksi* Aldrich, 1930	*Calodexia dives*	Neotropical	Panama	Rettenmeyer 1961a
*S. currani* Aldrich, 1930	*Calodexia agilis*, *C. dives*, *C. venteris*, *C. interrupta*	Neotropical	Panama	Rettenmeyer 1961a
*S. minuta* Townsend, 1897	*Calodexia agilis*, *C. fumosa*, *C. panamensis*, *Phasia ecitonis*	Neotropical	Panama	Rettenmeyer 1961a
'S. cf. ornatipes Kröber, 1914'	'*Chorisoneura* sp.'	Neotropical	Brazil	Lopes 1937
*S. speciosa* Aldrich, 1930	*Phasia ecitonis*	Neotropical	Panama	Rettenmeyer 1961a
*S. stylata* Townsend, 1897	'Orthoptera sp.', 'Blattodea sp.'^α^	Neotropical	Brazil	Lopes 1937
'*S.* sp.'	*Calodexia agilis*, *C. venteris*, *C. interrupta*	Neotropical	Panama	Rettenmeyer 1961a
*S. nitens* Brunetti, 1925	*Dichaetomyia pallidula*, *D. immaculiventris*, '*Phaonia* sp.'	Afrotropical	Ethiopia	Smith and Cunningham-Van Someren 1985
*S. nitens* Brunetti, 1925	'Suillia cf. acroleuca'^β^	Afrotropical	Nigeria	Smith and Cunningham-Van Someren 1985
*S. nitens* Brunetti, 1925	*Bengalia floccosa*, *B. peuhi*, *B. spinifemorata*, *Dichaetomyia albivitta*^γ^, *D. distanti*, *D. tristis*, *Hemigymnochaeta unicolor*, 'Isomyia cf. pubera', *Pseudohelina nigritarsis*^δ^, *Tricyclea bifrons*	Afrotropical	Kenya	Smith and Cunningham-Van Someren 1985
'S. cf. nitens Brunetti, 1925'	*Asarkina hulleyi*, 'Dichaetomyia n. sp. cf. mallochi', *D. serena*, *Dimorphia setulosa*, *D. tristis*, *Pyrellina chrysotelus*	Afrotropical	South Africa	Stuckenberg 1963
'S. cf. seguyi Camras, 1962'	'*Dichaetomyia* sp.'	Afrotropical	Madagascar	Smith 1967
'S. cf. seguyi Camras, 1962'	*Deltotus facetus*, *Helina carpiae*, *H. grisella*	Afrotropical	Madagascar	Couri and Barros 2010
'S. cf. seguyi Camras, 1962'	'Dichaetomyia (Panaga) sp.'	Afrotropical	South Africa	Couri and Barros 2010
*S. varifrons* Malloch, 1930	'Blattodea sp.', 'Blattella cf. lobiventris', 'Euloboptera cf. shelfordi'	Afrotropical	Kenya	Smith and Cunningham-Van Someren 1985
*S. westwoodi* Smith, 1967 ^ε^	'Gryllidae sp.', *Acanthogryllus fortipes*, '*Callogryllus* sp.', 'Pternoscirta cf. bimaculata', *Glossina morsitans*^ζ^	Afrotropical	Kenya Rwanda	Smith and Cunningham-Van Someren 1985, Berghe et al. 1956
*S. biannulata* (Say, 1823)	'*Gryllus* sp.', *G. rubens*	Nearctic	USA	Woodley and Judd 1998
*S. neglecta* Williston, 1883	*Oecanthus nigricornis*	Nearctic	USA	Etzler et al. 2020

**Table 4.** Number of *Stylogaster* eggs for specific body parts of calyptrate egg-carriers, summarised for each family and genus.

**Genus**	**Head**	**Thorax**	**Abdomen**	**Wing**	**Legs**	References
** MUSCIDAE **	45	193	60	8	4	
* Afromydaea *	2	1	-	-	-	Couri et al. 2013
* Coenosia *	-	1	-	-	-	Couri et al. 2013
* Deltotus *	1	6	3	-	-	Couri and Pont 2006, Couri and Barros 2010
* Dichaetomyia *	11	50	16	3	-	Stuckenberg 1963, Smith 1967, Couri and Pont 2006, Couri and Barros 2010, Couri et al. 2013
* Dimorphia *	-	14	5	1	-	Stuckenberg 1963, Couri et al. 2013
* Haematobosca *	-	3	1	-	-	Smith 1969
* Hebecnema *	-	-	1	-	-	Couri et al. 2013
* Helina *	-	11	1	-	-	Smith 1969, Couri and Barros 2010, Couri et al. 2013
* Limnophora *	-	1	2	-	-	Couri et al. 2013, Couri et al. 2019
* Musca *	1	2	1	-	-	Couri et al. 2019
* Neomyia *	-	3	-	-	-	Couri and Pont 2006, Couri et al. 2019
* Phaonia *	1	-	1	-	-	Smith 1969, Couri and Pont 2006
* Pseudohelina *	3	5	1	-	-	Couri et al. 2013, Couri et al. 2019
* Pyrellina *	2	11	3	-	-	Stuckenberg 1963, Couri et al. 2013
* Stomoxys *	24	85	25	4	4	Smith 1969, Couri et al. 2019
** CALLIPHORIDAE **	2	4	50	-	-	
*Bengalia*	1	1	-	-	-	Smith 1969
* Hemigymnochaeta *	1	1	1	-	-	Smith 1969
* Tricyclea *	-	3	49	-	-	Smith 1969, This study
** TACHINIDAE **	3	2	11	1	-	
* Phasia *	-	1	-	-	-	Rettenmeyer 1961a
* Calodexia *	3	1	11	1	-	Rettenmeyer 1961a

**Table 5.** Average number of *Stylogaster* eggs per fly for calyptrate egg-carriers, summarised for each family and genus. References as in Table 4.

**Genus**	**Average** **N^o^ of eggs**	**Range in** **N^o^ of eggs**	**Total N^o^ of eggs from all records**	**Total N^o^ of flies with eggs**
** MUSCIDAE **	1.42	1-6	310	218
* Afromydaea *	3	3	3	1
* Coenosia *	1	1	1	1
* Deltotus *	1.66	1-3	10	6
* Dichaetomyia *	1.45	1-4	80	55
* Dimorphia *	1.43	1-4	20	14
* Haematobosca *	1	1	4	4
* Hebecnema *	1	1	1	1
* Helina *	1.5	1-4	12	8
* Limnophora *	1.5	1-2	3	2
* Musca *	1	1	4	4
* Neomyia *	1.5	1-2	3	2
* Phaonia *	1	1	2	2
* Pseudohelina *	1.5	1-2	9	9
* Pyrellina *	1.5	1-2	16	12
* Stomoxys *	1.46	1-6	142	97
** CALLIPHORIDAE **	1.4	1-5	56	40
*Bengalia*	1	1	2	2
* Hemigymnochaeta *	1	1	3	3
* Tricyclea *	1.46	1-5	51	35
** TACHINIDAE **	1	1	17	17
* Phasia *	1	1	1	1
* Calodexia *	1	1	16	16

**Table 6.** Number of calyptrate specimens collected and number of specimens with *Stylogaster* eggs attached (proportions in parentheses). Overall parasitism rate from the only known hosts (underlined). Taxa not identified to species level follow the original identification and are put in quotation marks, for example, '*Dichaetomyia* sp. 1' from Smith (1967). [*The numbers from Woodley and Judd (1998) are estimates, as they do not give precise numbers for the hosts and only record the number of *Stylogaster* puparia].

**Number of specimens collected per species**					**Specimens with Stylogaster eggs attached**			
**Species**	**Country**	**Males**	**Females**	**Total**	**Males**	**Females**	**Total**	**References**
** CALLIPHORIDAE **								
*Tricyclea fasciata*	Tanzania	29	10	39	3 (10.3%)	6 (60.0%)	9 (23.0%)	This study
*Tricyclea* sp. A	Tanzania	26	68	94	8 (30.8%)	15 (22.1%)	23 (24.4%)	This study
** MUSCIDAE **								
'*Dichaetomyia* sp. 1'	Madagascar	61	52	113	2 (3.3%)	5 (9.6%)	7 (6.2%)	Smith 1967
'*Dichaetomyia* sp. 2'	Madagascar	40	50	90	6 (15%)	5 (10%)	11 (12.2%)	Smith 1967
*Dimorphia* spp.	South Africa	-	21	21	-	9	9 (42.9%)	Stuckenberg 1963
Muscidae spp.	Ethiopia	-	-	908			89 (9.8%)	Couri et al. 2019
** TACHINIDAE **								
*Calodexia* spp.	Panama	-	-	1802	-	15	15 (0.8%)	Rettenmeyer 1961a
*Phasia ecitonis*	Panama	-	-	531	1	1	2 (0.4%)	Rettenmeyer 1961a
** ORTHOPTERA **								
*Gryllus rubens*	USA	-	-	1000*	-	-	25-30* (2.5-3%)	Woodley and Judd 1998
*Oecanthus nigricornis*	USA	-	-	674	-	-	149 (22%)	Etzler et al. 2020
