# Supplementary material for: Stylogaster eggs on blow flies attracted to millipede defence secretions in Tanzania, with a stab at summarising their biology (Diptera: Conopidae & Calliphoridae)
Source: Biodivers Data J. 2020 Jun 30;8:e54808. doi: 10.3897/BDJ.8.e54808 (PMC7340630; doi:10.3897/BDJ.8.e54808)
Supplement: Supplementary material 1 — Supplementary Table S1 [file bdj-08-e54808-s001.pdf]

Stylogaster eggs on blow flies attracted to millipede defense secretions in Tanzania, with a stab at summarizing the biology (Diptera: Conopidae & Calliphoridae)

Biodiversity Data Journal

Arn Rytter Jensen, Sapienza University of Rome, Rome, Italy and Natural History Museum of Denmark, University of Copenhagen, Copenhagen, Denmark

Freja Odgaard, Natural History Museum of Denmark, University of Copenhagen, Copenhagen, Denmark

Pierfilippo Cerretti, Sapienza University of Rome, Rome, Italy

Thomas Pape, Natural History Museum of Denmark, University of Copenhagen, Copenhagen, Denmark

Supplementary Materials.

Supplementary Table 1. Placement and number of *Stylogaster* eggs for each individual fly examined. The ID of the fly corresponds to the visual representation of this data in Fig. 2.

ID of fly	Species	Sex	*Stylogaster* eggs	Sternite 5	Tergite 1+2	Tergite 3	Tergite 4	Tergite 5	Tergite6	TST7+8	Epandrium	Proepisternum
**A**	*T.* n. sp.	male	1							1		
**B**	*T.* n. sp.	female	1	1								
**C**	*T.* n. sp.	male	1					1				
**D**	*T.* n. sp.	female	1				1					
**E**	*T. fasciata*	female	1							1		
**F**	*T. fasciata*	male	2			1		1				
**G**	*T.* n. sp.	female	1								1	
**H**	*T. fasciata*	female	1							1		
**I**	*T.* n. sp.	male	2					1	1			
**J**	*T.* n. sp.	male	5				1	1	3			
**K**	*T.* n. sp.	male	1						1			
**L**	*T. fasciata*	male	1						1			
**M**	*T.* n. sp.	female	2					1	1			
**N**	*T. fasciata*	female	2			1		1				
**O**	*T. fasciata*	female	4					2			2	
**P**	*T. fasciata*	female	1					1				
**Q**	*T.* n. sp.	male	1						1			
**R**	*T.* n. sp.	female	1				1					
**S**	*T.* n. sp.	female	1				1					
**T**	*T.* n. sp.	female	2						1	1		
**U**	*T.* n. sp.	female	1						1			
**V**	*T.* n. sp.	female	1					1				
**X**	*T.* n. sp.	female	1					1				
**Y**	*T.* n. sp.	female	1					1				
**Z**	*T. fasciata*	female	1				1					
**A1**	*T.* n. sp.	female	1						1			
**B1**	*T.* n. sp.	female	2						1			1
**C1**	*T. fasciata*	male	1					1				
**D1**	*T.* n. sp.	male	2					1	1			
**E1**	*T.* n. sp.	female	2				1	1				
**F1**	*T.* n. sp.	female	2					2				
**G1**	*T.* n. sp.	male	1					1				
**Total**	32		48	1	0	2	6	18	13	4	3	1
